# The Sydney triage to admission risk tool (START) to improve patient flow in an emergency department: a model of care implementation pilot study

**DOI:** 10.1186/s12873-019-0290-x

**Published:** 2019-12-05

**Authors:** Anja Ebker-White, Kendall J. Bein, Saartje Berendsen Russell, Michael M. Dinh

**Affiliations:** 1 Emergency Department, Royal Prince Alfred Hospital, Missenden Road, Camperdown NSW, Sydney, 2050 Australia; 2 RPA Green Light Institute, Royal Prince Alfred Hospital, Missenden Road, Camperdown, NSW 2050 Australia

**Keywords:** Emergency, Performance, Length-of-stay

## Abstract

**Background:**

The Sydney Triage to Admission Risk Tool (START) is a validated clinical analytics tool designed to estimate the probability of in-patient admission based on Emergency Department triage characteristics.

**Methods:**

This was a single centre pilot implementation study using a matched case control sample of patients assessed at ED triage. Patients in the intervention group were identified at triage by the START tool as likely requiring in-patient admission and briefly assessed by an ED Consultant. Bed management were notified of these patients and their likely admitting team based on senior early assessment. Matched controls were identified on the same day of presentation if they were admitted to the same in-patient teams as patients in the intervention group and same START score category. Outcomes were ED length of stay and proportion of patients correctly classified as an in-patient admission by the START tool.

**Results:**

One hundred and thirteen patients were assessed using the START-based model of care. When compared with matched control patients, this intervention model of care was associated with a significant reduction in ED length of stay [301 min (IQR 225–397) versus 423 min (IQR 297–587) *p* < 0.001] and proportion of patients meeting 4 h length of stay thresholds increased from 24 to 45% (*p* < 0.001).

**Conclusion:**

In this small pilot implementation study, the START tool, when used in conjunction with senior early assessment was associated with a reduction in ED length of stay. Further controlled studies are now underway to further examine its utility across other ED settings.

## Background

Emergency Departments (ED) continue to face significant challenges related to overcrowding and access block [1, 2]. Optimising patient flow under these conditions requires both safe and efficient patient management in ED as well as supportive hospital bed management. Several strategies that have been reported to improve patient throughput include streaming units within ED, physician assisted triage, and senior early assessment [3–6]. In these models of care, senior ED clinicians briefly assess patients shortly after arrival and expedite earlier investigations and appropriate treatment. Such models of care will become increasingly important with the rise in ED presentations in older patient and those with complex comorbidities, where care is coordinated by defined in-patient services and specialists [7].

We have previously described a clinical analytics tool to support early streaming and senior early assessment, called the Sydney Triage to Admission Risk Tool (START) [8]. In brief, START was a risk score calculated based on the weighted sum of a number of variables including age, mode of arrival, presenting problem and triage category to derive a probability of in-patient admission. It was derived using state-wide emergency data from New South Wales Australia, validated prospectively [8–10] and evaluated using machine learning techniques [11]. START aimed to improve patient flow by supporting earlier disposition decision making by senior ED clinicians and flagging the need for an in-patient bed at an earlier stage in the ED assessment process providing the basis for expedited bed management strategies. The START scoring system is attached as Appendix 1. The aim of the present study was to pilot the START tool in combination with a senior early assessment model of care and determine its effectiveness with respect to ED length of stay in a sample of patients who were likely to require in-patient admission based on the START calculation. The evaluation will provide important information regarding the feasibility of the tool and how it could be further evaluated in other settings.

## Methods

### Aim

To determine the impact of using the Sydney Triage to Admission Risk Tool (START) in combination with senior early assessment compared with senior early assessment alone, with respect to ED length of stay and ED performance.

### Design

This was a single centre pilot study of a model of care implementation.

### Setting

The study was conducted at an inner city tertiary level hospital Emergency Department in metropolitan Sydney, Australia that sees around 80,000 presentations annually. Tertiary level hospitals in Australia provide the highest level of specialist care and resources. Senior early assessment is currently the model of care for patients presenting to the ED at this institution where the patient is initially assessed by the most senior clinician available in ED. From 0800 to midnight, tertiary hospitals EDs are staffed by board certified emergency physicians.

### Patient population

As this was a pilot study, patients were derived from a convenience sample of adult patients presenting to the ED during business hours on Monday and Tuesdays between September and November 2018, who were assessed at triage and identified using the START tool as likely requiring in-patient admission (START score > 16). These times were chosen as they represent the busiest weekday time periods of the week with respect to in-patient presentations to ED.

### Intervention

As senior early assessment and team-based models of care were already in place at this institution, implementation and engagement of the START tool consisted only of a group email to ED Consultants and a 5 min introduction at the start of a shift outlining the proposed intervention.

The START tool was applied at the point of triage on patients by a single investigator to determine the likelihood of in-patient admission. This occurred in parallel with usual triage practices. The same investigator then alerted the ED Consultant in charge of any patients who were assessed as likely requiring an in-patient admission (START score > 16 points). The role of the ED Consultant was to decide based on the senior early assessment and START score whether the patient would in fact be admitted and which in-patient specialist service the patient would be most likely admitted under. The ED Navigator, a senior nurse acting as a bed liaison officer [4] would then communicate this decision to hospital bed managers and allocate or search for a bed based on this information whilst full assessment including investigations, consultations and initial management were completed in ED.

### Controls

Matched controls were randomly identified through the ED tracking system, and comprised patients, who presented on the same date (nearest to time of presentation before or after intervention case), with the same START score category and were admitted under the same clinical service as intervention patients. Therefore this pilot study compared patients who were admitted using the START-assisted model of care with a control group of patients who were admitted under standard practices.

### Study outcomes

The primary outcomes were length of stay for admitted patients and the proportion of admitted patients with an ED length of stay less than 4 h. The secondary outcomes were time to final disposition, defined by the time that “admission ready” icons were fired in the ED tracking system and the proportion of intervention patients who were admitted to an inpatient unit (including short stay unit admissions).

### Data collection and statistical analyses

Variables collected through existing patient information systems included age, gender, triage category, ED length of stay, disposition and admitting specialty. Wilcoxon sign rank tests were used to compare matched length of stay (in minutes) between groups and McNemar’s test used to compare proportions of patients meeting 4 h benchmarks (Emergency Treatment Performance or ETP). The odds of patients meeting the 4 h ED length of stay benchmark were adjusted for any observed between-group differences using multivariable conditional logistic regression to account for matched data. As this was a pilot study, a priori sample size calculation was not performed.

## Results

### Study population

During the study period 155 patients were initially identified using the START tool at triage as likely requiring in-patient admission and of these 113 (78.7%) patients were assessed by the ED Consultant in charge and an admission disposition and clinical specialty assigned based on the START score and initial assessment. The most common reasons for non-assessment by the ED Consultant in charge were lack of availability (*n* = 30) and disagreement regarding the initial START score recommendation (*n* = 12). Baseline characteristics were compared between intervention and matched control groups in Table 1. Of the 224 admitted patients in both intervention and control groups, the most common admission specialties were geriatrics (26.4%), cardiology (12.1%), respiratory (9.4%) and neurology (7.7%). Six patients (all in the intervention group) were admitted to the ED Short Stay Unit located separately to ED.

**Table 1:** Baseline characteristics of cases undergoing senior early assessment with the aid of the Sydney Triage to Admission Tool (START) (intervention) and control groups IQR = Interquartile Range

Variable	Intervention	Control	*P* value
	*N* = 113	*N* = 113	
Age (mean SD) years	68.6 (18.8)	67.2 (20.3)	0.57
Male (%)	61 (54.0)	54 (47.8)	0.35
Triage category (%)			0.27
2	42 (36.4)	33 (29.2)	
3	68 (60.2)	71 (62.8)	
4	3 (2.7)	9 (7.4)	
Ambulance arrival	70 (62.0)	60 (53.1)	0.18
START score (median IQR)	26 (22–30)	24 (19–29)	0.01
In-patient admission	109 (96.5)	106 (93.8)	0.35

### Outcomes

The intervention group was associated with a significantly reduced length of stay compared to controls (Table 2) [301 min (IQR 225–397) versus 423 min (IQR 297–587) *p* < 0.001] and proportion of patients meeting 4 h length of stay thresholds increased from 21.0 to 39.8% (*p* < 0.001). When the 42 patients who were scored as likely admission using the START tool but no evaluated using senior early assessment were included in the intervention group the median ED length of stay was still lower than the control group (308 min IQR 217–410, *p* < 0.001).

**Table 2:** Study outcomes for patients *Admitted patients only

Outcome	Intervention *N* = 113	Control *N* = 113	*P* value
ED length of stay* (mins) median IQR	301 (225–397)	423 (297–587)	< 0.001
Time to disposition (mins median IQR)	32 (21–50)	164 (104–219)	< 0.001
ED Length of stay < 4 h (%)	45 (39.8)	24 (21.2)	< 0.001

The difference in ED length of stay remained statistically significant (*p* < 0.001) even after excluding the six intervention patients who were admitted to the ED short stay unit. After adjusting for differences in START scores between the control and intervention groups and stratified with matched pairs using conditional logistic regression, the odds of meeting four hour length of stay threshold was around two times higher in the intervention compared to control groups (OR 2.6 95%CI 1.4, 5.2 *p* = 0.003).

When all patients assessed using the START tool as being likely admission were evaluated 139 out of 155 (90%) were correctly classified as an in-patient admission. Of the 113 patients who were in the intervention group (i.e. scored using START and assessed by the ED Consultant), 96% were correctly classified as an admission and 11 (9%) patients had the admitting specialty team that was changed prior to transfer to in-patient wards.

A post hoc analysis of control group patients who presented after business hours (0800–1700) did not reveal a longer length of stay compared to those presenting in-hours [In hours median time 481 min (IQR 311–953) versus after hours 462 min (IQR 252–656) *p* = 0.42].

There were no adverse events or issues raised by in-patient teams resulting from the pilot intervention.

## Discussion

The present study was conducted to evaluate a pilot implementation of a model of care designed to facilitate patient flow in ED. The model of care comprised senior early assessment in ED supported by a validated clinical analytics tool called START. The tool was used to support senior early decision making by alerting senior ED clinicians to appropriate patients for whom in-patient admission was likely and bed management facilitated at an earlier stage. The key driver of the study was to assist with engagement and confidence of senior clinicians and hospital administration in the model of care prior to formal implementation and more controlled studies, and the present study provides important insights in this regard.

The present study demonstrated that over 90% of patients assessed using the START tool as likely requiring an admission and briefly assessed by the ED Consultant were in fact admitted. In such patients, the median length of stay was just under 3 h less than control patients, contributing to a marked improvement in the proportion of intervention group patients meeting the 4 h ED length of stay benchmark. These initial findings provide the basis to evaluate this model of care further determine its effectiveness and translate it across other settings.

There are a number of important implications for these findings. Firstly, although there are numerous analytics and risk prediction tools in use clinically, [12–14] and several that have reported admission prediction models, [15–18] this is the first initiative in the Australian context to demonstrate performance improvements associated with real-time use of a clinical analytics tool. It should be noted that the tool itself was not designed to directly alter patient flow. Rather, it was the use of this tool to support senior early assessment and identify appropriate patients who require in-patient admission prior to completion of ED assessment, which provided the basis for expedited bed management strategies. The basic premise of the model of care was that earlier and data-driven disposition making would assist clinicians and bed managers to locate an appropriate inpatient ward bed earlier in the ED encounter, making it more likely that the bed would be ready by the time ED assessment and treatment were complete. This is in contrast to the current model of care whereby patient assessment is completed and care “accepted” by in-patient teams before the bed finding process is initiated. Under the proposed model, in-patient bed finding would commence several hours earlier and expedited particularly for patients with chronic medical conditions.

Secondly, the model of care provides an example of the effectiveness of patient flow strategies when supported by hospital and bed managers. Previous studies of senior early assessment in ED, which included ED Navigators and other bed management strategies at this institution showed no improvement in the proportion of admitted patients meeting the 4 h length of stay benchmark, which remained around 20% [19]. These were consistent with the length of stay in the control group reported this study, and current performance data at this institution. The present study extended this model to include not just a clinical analytics tool assisted senior early assessment, but also the finding and allocation of beds based on senior early assessment. There will of course be many times, during periods of access block, when an appropriate bed will not be available regardless of the lead time provided through senior early assessment, but on average, providing earlier notification of the need for an in-patient bed was shown to reduce overall ED length of stay.

There are several acknowledged limitations to this study. This was a relatively small single centre pilot study using a tool that was developed by the investigators at this site. The study was designed to reflect how the tool would be implemented in clinical practice. In this case, a sample of patients was selected based on the likelihood of in-patient admission using the START tool and compared to matched controls based on day of presentation and admitting in-patient service. Matching introduced the potential for selection bias, and this was minimised by randomly selecting matched controls on the basis of arrival date and time, START score, age and admission specialty. In addition, the length of stay and ETP performance in the control group was consistent with current data on in-patient admission and ED length of stays at this institution, suggesting that selection bias was not a substantial factor in this study [19]. Finally, we did not examine hospital length of stay or readmissions to hospital and this would be a useful measure for future implementation studies.

Given the findings of the present study, it would be important for this model of care to be evaluated more rigorously using either a patient level randomised control study or cluster randomised control study at a number of different sites to confirm external validity. A randomised control trial may be difficult to perform in this context given the likelihood of crossover contamination, but the lessons from this pilot will be used in a forthcoming and ethics approved implementation trial using a randomised control design. Efforts are also now underway to fund a translational research study to implement this at scale, and further refine the tool using machine learning with linked datasets.

Finally, the study was conducted only on Mondays and Tuesdays, as these were high demand times with respect to inpatient admissions and the tool is designed to assist with the flow of inpatients through the ED. Uncontrolled confounders include workflow styles of particular consultants and openness to model of care changes. Mondays and Tuesdays were also the busiest and challenging days of the week in terms of patient volume and access block, which would be expected to bias results towards the null hypothesis. Differences in time of day of presentation in control group patients may have accounted some of the differences in ED length of stay, however in this study, control patients presenting after hours were not associated with a longer length of stay compared to control patients presenting during business hours.

Further challenges include the incorporation of the START score into existing patient information systems. As most of the variables that comprise the START score can be automatically calculated or observed at the point of triage, opportunities exist for this clinical analytics tool to be displayed in real time within existing electronic patient information systems. Adjustments to the triage process may need to be required to accommodate this and further studies are required to determine which groups of patients would benefit most from the additional intervention required at triage.

## Conclusion

In conclusion, in a limited pilot implementation study, a clinical analytics tool to support senior early assessment in ED was associated with reduced ED length of stay. These preliminary results provide the basis to further study the implementation and evaluation of this tool using larger studies.

## Supplementary information

**Additional file 1.** Start Tool.

## Data Availability

The datasets used and/or analysed during the current study are available from the corresponding author on reasonable request.

## Abbreviations

ED: Emergency department
START: Sydney Triage to Admission Risk Tool
