# Supplementary material for: The Sydney triage to admission risk tool (START) to improve patient flow in an emergency department: a model of care implementation pilot study
Source: BMC Emerg Med. 2019 Dec 5;19:79. doi: 10.1186/s12873-019-0290-x (PMC6896669; doi:10.1186/s12873-019-0290-x)
Supplement: Supplementary file 1 — Additional file 1. Start Tool. [file 12873_2019_290_MOESM1_ESM.docx]

**START TOOL**

Pt Sticker

| **Variable** | **Risk score** |
| --- | --- |
| **Age** |  |
| 16-19 yrs | 0 |
| 20-39 yrs | +1 |
| 40-59 yrs | +3 |
| 60-79yrs | +6 |
| ≥80 yrs | +9 |
| **Ambulance arrival** | +4 |
| **Triage category** |  |
| 1 | +24 |
| 2 | +16 |
| 3 | +11 |
| 4 | +5 |
| 5 | 0 |
| **Admission within 30 days** | +3 |
| **Hour of presentation** |  |
| 0800-1759 | +1 |
| 1800-2259 | 0 |
| 2300-0759 | 0 |
| **Presenting problem** |  |
| Abdominal, gastrointestinal | +2 |
| Cardiovascular | -3 |
| General symptoms | 0 |
| Febrile illness | +3 |
| Injury | -4 |
| Respiratory | 0 |
| Musculoskeletal | -3 |
| Neurological | -1 |
| Mental health | -2 |
| Toxicological | -2 |
| ENT/eye/head and neck | -6 |
| Administrative | -3 |
| Genitourinary | -1 |
| Social | +1 |
| Endocrine | 0 |
| Obstetrics, Gynaecology | -3 |
| Skin, allergy | -2 |
| Other medical | +5 |
| **3 assumed presentations in last week** | Y / N |
| **Decreased mobility or frailty** | Y / N |
| **Multiple or Major co-morbidities** | Y / N |
| **Referred by GP/Specialist with diagnosis and or needing admission** | Y / N |
| **Significant ED overcrowding (>3 ambulances or TOC trolleys waiting to offload or >25 patients waiting to be seen)** | Y / N |
| **Other factors....please specify** | ...............................................................  ............................................................... |
| **TOTAL START SCORE** |  |

**Recommendation based on risk score only. Further clinical assessment and clinical judgement required prior to final disposition decision.**

|  | **Risk Score Range** | **Stream/ Location** |
| --- | --- | --- |
| **Very Likely Discharge** | < 5 | WR / RAFT/refer to Ambulatory care or HITH |
| **Likely Discharge** | 5-10 | RAFT / EMU results/transport pending |
| **Indeterminate** | 11-15 | Decision pending clinician assessment |
| **Likely Admission** | 16-25 | Look for inpatient bed, work-up for admission and notify ED Admitting Officer and NUM |
| **Very Likely Admission** | > 25 | Book inpatient bed and inform bed management and in-patient team of arrival |

**Examples of major co-morbidities**

On chemotherapy/cancer treatment

Renal or liver transplant or haemodialysis

Congenital heart disease

Cystic fibrosis
